# Genome-wide eQTLs and heritability for gene expression traits in unrelated individuals

**DOI:** 10.1186/1471-2164-15-13

**Published:** 2014-01-09

**Authors:** Shengjie Yang, Yiyuan Liu, Ning Jiang, Jing Chen, Lindsey Leach, Zewei Luo, Minghui Wang

**Affiliations:** Department of Biostatistics and Computational Biology, School of Life Sciences, Laboratory of Population & Quantitative Genetics, State Key Laboratory of Genetic Engineering, Fudan University, Shanghai, 200433 China; School of Biosciences, The University of Birmingham, Edgbaston, Birmingham, B15 2TT UK; Division of Cardiovascular & Diabetes Medicine, University of Dundee, Dundee, DD1 9SY UK

**Keywords:** Microarray gene expression, eQTLs, Heritability, Mixed model, HapMap populations, Epistasis

## Abstract

**Background:**

While the possible sources underlying the so-called ‘missing heritability’ evident in current genome-wide association studies (GWAS) of complex traits have been actively pursued in recent years, resolving this mystery remains a challenging task. Studying heritability of genome-wide gene expression traits can shed light on the goal of understanding the relationship between phenotype and genotype. Here we used microarray gene expression measurements of lymphoblastoid cell lines and genome-wide SNP genotype data from 210 HapMap individuals to examine the heritability of gene expression traits.

**Results:**

Heritability levels for expression of 10,720 genes were estimated by applying variance component model analyses and 1,043 expression quantitative loci (eQTLs) were detected. Our results indicate that gene expression traits display a bimodal distribution of heritability, one peak close to 0% and the other summit approaching 100%. Such a pattern of the within-population variability of gene expression heritability is common among different HapMap populations of unrelated individuals but different from that obtained in the CEU and YRI trio samples. Higher heritability levels are shown by housekeeping genes and genes associated with *cis* eQTLs. Both *cis* and *trans* eQTLs make comparable cumulative contributions to the heritability. Finally, we modelled gene-gene interactions (epistasis) for genes with multiple eQTLs and revealed that epistasis was not prevailing in all genes but made a substantial contribution in explaining total heritability for some genes analysed.

**Conclusions:**

We utilised a mixed effect model analysis for estimating genetic components from population based samples. On basis of analyses of genome-wide gene expression from four HapMap populations, we demonstrated detailed exploitation of the distribution of genetic heritabilities for expression traits from different populations, and highlighted the importance of studying interaction at the gene expression level as an important source of variation underlying missing heritability.

**Electronic supplementary material:**

The online version of this article (doi:10.1186/1471-2164-15-13) contains supplementary material, which is available to authorized users.

## Background

In genome-wide association studies (GWAS) of conventional complex traits such as human complex diseases, a fundamental and yet unsolved question is that of so-called missing heritability, *i.e.*, the significant and often numerous variants collectively explaining only a small fraction of the total phenotypic variation [1, 2]. For example, recent studies show that ~50 variants explain only ~5% of the phenotypic variation for human height, a highly heritable trait with narrow sense heritability of ~80% [3, 4]. While fully resolving the missing heritability remains a challenging task, we have studied the heritability of gene expression traits to shed light on the relationship between trait phenotypic variation and genetic variation on the basis that gene expression is the process linking genetic information to the final phenotype, and is itself genetically controlled. Furthermore, gene expression is generally assayed in well controlled experiments, suggesting less vulnerable to environmental variation than conventional phenotypes, and is thus an ideal choice for studying the extent to which genetic components contribute to phenotypic variation.

With the advent of DNA microarrays and more recently deep sequencing-based profiling approaches, the expression of thousands of genes can be readily measured simultaneously, creating a global snapshot of cellular activity. A number of studies have assessed the heritability of microarray gene expression traits in different species, including *Arabidopsis*[5] and rat [6]. The heritability of gene expression means it can be subject to the same quantitative trait loci (QTL) analyses as conventional trait data to reveal the so-called expression QTLs (eQTLs). For example, several studies have analysed the gene expression profile of lymphoblastoid cell lines (LCLs) from HapMap samples and reported that genetic factors make an important contribution to variation in gene expression [7–11]. These studies, however, focused on differentially expressed genes and exploring *cis* and *trans* genetic determinants of gene regulations either from one single ethnic group or across ethnic groups. There is not yet any report in the literature on the gnome-wide distribution of heritabilities of gene expression traits and the *cis* and *trans* eQTLs across different HapMap populations. Furthermore, the phenotypic variations explained by interactions between eQTLs have never been exploited at a genome-wide scale in humans to our best knowledge. Recently, Price et al. [12] analysed microarray gene expression data from blood and adipose samples of Icelandic family cohorts and began to partition the heritability into *cis* and *trans* components using a variance component model composed of polygenic effects estimated using identity by descent (IBD) for chromosome segments both proximal (*cis*) and distal (*trans*) to the gene of interest. However, their method implicitly assumed the sum of variance components to be unity after normalising gene expression values to have mean 0 and variance 1, and only genetic variance component parameters were estimated using a binary search algorithm. While samples of related individuals were collected and hence genetic correlations among samples were expected, assumption of unity variance virtually neglected the variance-covariance structure and hence might introduce bias in the estimation of heritability. Moreover, a large number of negative heritability estimates were derived in that study, raising the challenge for a meaningful biological explanation of the negative heritability estimates. While it is possible that variation noise caused the negative estimates of heritabilities as discussed in Price et al. [12], a robust statistical approach which enables to prevent such negative heritability estimate is highly desirable.

In this study, we re-analysed gene expression microarray data from four HapMap populations using a statistically rigorous variance component model with motivation to explore (1) the global pattern of the distribution of heritabilities of gene expression traits in unrelated individuals, (2) the cumulative genetic contribution of *cis* and *trans* acting eQTLs to gene expression heritability, and (3) the potential of gene-gene interactions in explaining the missing heritability at gene expression level.

## Methods

### Gene expression data and quality control

We analyzed the gene expression levels measured previously in LCLs from 210 unrelated HapMap individuals, using Illumina’s human whole-genome expression array (WG-6 version 1) [9]. In this experiment, each of the two *in vitro* transcription (IVT) reactions from the 210 samples was hybridized to each of two arrays, resulting in four replicate hybridizations for every sample. We downloaded background-corrected gene expression values from the Gene Expression Omnibus (GEO) database (accession number GSE6536), and then carried out quantile normalisation across replicates of a single individual and subsequently median normalisation across all individuals by using the R package beadarray [13]. It should be noted that the Illumina Genome Studio software can work out detection scores for each probe and flag the presence/absence calls of expression of the features. However, the detection scores were not provided for the current microarray dataset and hence we applied a filter to exclude the microarray probes based on their expression levels as described below.

### Selecting probes

We conducted BLAT analysis [14] to map all 47,294 Illumina array probes onto human cDNA sequences from Ensembl (hg19). Among these, 21,152 mapped probes were retained after removing probes mapped with over 90% identity to multiple genes or mapped to sex chromosomes or mitochondrial DNA. Further removed were 24 probes that carried at least one genetic variant within the probe region (according to the Ensembl Variation database). This further reduced the potential bias in gene expression estimation due to the mis-match between probe and transcript sequence. To exclude those genes with an extremely low expression level, additionally, we filtered out those probe features whose raw intensity values were smaller than background noises in more than half of the total individuals in all the four replicated arrays, resulting in 12,158 probes from 10,720 genes which were recognized to be expressed features or genes. Finally, for the genes surrogated by multiple probes, we took the average over all relevant probes as estimates of their expression levels. Following heritability and eQTL analyses were based on the 10,720 expressed genes.

### Genotype data and quality control

We downloaded the phase II and III combined genotype data for HapMap individuals from the HapMap project website. In total, there are over 4 million SNPs genotyped for the present HapMap samples. Genotype quality checking was performed independently in each individual population. SNPs were removed for each of the HapMap populations if they were: i) located on sex chromosomes, ii) genotyped in less than 90% of individuals, iii) with allele frequency < 0.05, and/or iv) demonstrating significant departure from Hardy-Weinberg Equilibrium (*P* < 0.001). The final dataset contained genotypes at 1,299,240 consensus SNPs from all four HapMap populations.

### Genetic relationship estimation

To estimate heritability from fitting a variance component model, it is firstly necessary to estimate pairwise genetic relationship coefficients for the HapMap samples. A number of statistical methods have been proposed for estimating genetic relationships from genome-wide high density marker genotypes for homogeneous (*i.e.*, non-stratified) populations, e.g. the program PLINK [15]. However, for the present analysis using samples from four HapMap populations, relationship estimation will be biased unless the population structure is considered. Since the population origin of each HapMap individual is clear and the different populations have been geographically isolated from each other for many generations, it is reasonable to assume that individuals from different populations are unrelated and their relationship coefficients are zero. Therefore, to adjust for population stratification we used PLINK to estimate the coefficients of genetic relationship based on autosomal marker genotypes in each HapMap population independently and then merged all four population genetic relationship matrices by setting the relationship coefficients between individuals from different populations to be zero. To account for linkage disequilibrium (LD) between SNPs, we utilized the PLINK SNP pruning function to generate a subset of 36,609 SNPs that were in approximate linkage equilibrium (pairwise genotypic correlation *r*^2^ < 0.05) to be used for relationship estimations. It should be noted that the genetic relationship coefficients derived from PLINK are the probabilities of genome-wide allelic identical by descent (IBD) [15]. In the following, we denote by **K**_*pop*_ as the genetic relationship matrix in population *pop* (where *pop* = CHB, JPT, CEU or YRI) and **K** as the final merged genetic relationship matrix.

### Gene expression heritability estimation

Let *y*_*ij*_ be the normalised gene expression level for gene *g* on the *j*th array for the *i*th individual. We use the following linear mixed model to estimate gene expression heritability
1

where *μ* is the model intercept, **S**_*i*_ is a row vector of non-genetic covariates with coefficients *β*, *u*_*i*_ is the polygenic effect, and *e*_*ij*_ is the random residual term. Here **S**_*i*_ indexes the population origin for individual *i*, to correct for the population structure embedded in the HapMap samples. It should be noted that an intercept term is included in the model and hence indexing to one of the four populations is omitted in **S**_*i*_ to avoid singularity of the model design matrix. Assume *u* ~ *N*(0, ) and *e* ~ *N*(0, ), where  is the variance of the polygenic effect, **K** is the merged genetic relationship matrix,  is the residual variance, and **I** is a an identity matrix. Assuming zero covariance among random factors *u*_*i*_ and *e*_*ij*_, the overall phenotypic variance-covariance of the gene expression traits can be expressed as
2

where **Z** is a design matrix relating gene expression levels from each array to individual sample. We implemented a restricted maximum likelihood (REML) approach using R (http://www.r-project.org/) programming language to obtain the maximum likelihood estimates (MLEs) of the model parameters [16]. With the estimates of variance components*, i.e.*,  and , narrow sense heritability of the gene expression trait can be estimated by . Gene expression heritability analysis was performed in the four HapMap populations combined, and also in each individual HapMap population using a simplified linear mixed model *y*_*ij*_ = *μ* + *u*_*i*_ + *e*_*ij*_, where *u* ~ *N*(0, ) and *e* ~ *N*(0, ).

### eQTL scan and eQTL heritability estimation

To scan for genome-wide eQTLs for each gene, we averaged the normalised expression levels for each gene from four replicated arrays for each individual and then tested for gene-SNP associations with correction for population structure as in the following model
3

where *y*_*i* ·_ is the mean expression value for a target gene for individual *i*, **x**_*ik*_ is the genotype score of individual *i* at the *k*th SNP marker, with values 0, 1 and 2 representing the number of a reference allele at the SNP locus, *α*_*k*_ is the regression coefficient at the SNP, and *e*_*i*_ is the residual term. *μ*, **S**_*i*_ and *β* are defined as in equation (1). A *t*-test is then carried out against the null hypothesis of *α*_*k*_ = 0 for the target gene at the *k*th SNP. A conservative Bonferroni *P* value threshold (0.05/1,299,240 = 3.85 × 10^-8^) was applied to account for the large number of tests. Significant SNPs were merged into eQTLs using criteria as detailed in Results.

For gene-SNP associations surpassing the Bonferroni *P* value threshold, we were interested in how much of the genetic variation for the gene expression trait could be explained by the significant SNPs. For this purpose, we extended the above mixed model (1) by incorporating a SNP effect term as
4

where *u* ~ *N*(0, ), *e* ~ *N*(0, ), and all variables are defined as per equations (1) and (3). Again, the REML approach is applied to obtain the MLEs of the two variance components  and . The model expressed in equation (4) is essentially the same as the variance component models defined previously by Yu et al. [17] and Kang et al. [18]. Denoting by  and  the estimates of variance components from equation (1) and  and  the corresponding estimates from equation (4), the estimate of the phenotypic variance explained by a test SNP can be approximated by . Then, the proportion of phenotypic variance of the gene expression trait accounted for by a test SNP can be approximately formulated as
5

This single point heritability estimation approach can be readily applied to estimate aggregated heritability from multiple SNPs by simply fitting multiple SNP genotypes into the above equation (4).

## Results

### Population structure and genetic relationship

We firstly utilized multidimensional scaling (MDS) within the PLINK program [15] to investigate population structure among the four HapMap populations (CHB, JPT, CEU and YRI) based on marker genotype data. The first two principal coordinates (PCo) clearly separate the CEU and YRI populations from each other and from the two Asian populations (CHB and JPT), which are in turn distinguished from each other by the third PCo (Additional file 1). Separation of the four geographically isolated HapMap populations indicates it would not be appropriate to treat the four populations as a homogeneous sample, but instead there should be proper control of the heterogeneity caused by the population structure effect in both the genetic relationship inference and association analysis.

We used PLINK to infer the genetic relationship matrix for each of the four HapMap populations independently and then merged the resulting four matrices by setting inter-population pairwise relationship coefficients to zero. The mean (and standard deviation) of IBD coefficients were 0.0067 (0.0074), 0.0056 (0.0073), 0.0068 (0.0080), and 0.0035 (0.0145) for CHB, JPT, CEU and YRI HapMap populations, respectively. Clearly the IBD coefficients were very low, as expected since individuals collected in each population were genetically unrelated. Consistent with previous reports (e.g., [19]), six pairs of individuals were evidently highly related (IBD coefficient > 0.05). Three related pairs were from the YRI population: NA18913 and NA19238 (IBD coefficient 0.5005), NA19130 and NA19192 (IBD coefficient 0.2392), and NA19092 and NA19101 (IBD coefficient 0.1231). The remaining three potentially related pairs were between CEU individuals NA06993 and NA07022 (IBD coefficient 0.0696), NA06993 and NA07056 (IBD coefficient 0.0686), and NA12155 and NA12264 (IBD coefficient 0.0679). To avoid having close relatives in the data, we selectively excluded one individual with greater number of missing genotypes for each of the three pairs of highly related individuals.

### Gene expression heritability

Normalised gene expression levels were fitted into the variance component model (equations 1 and 2) to estimate the proportion of phenotypic variance explained by polygenic effects, *i.e.*, the heritability in the present HapMap populations. A total of 10,720 genes were selected for heritability estimation using the REML technique. After REML fitting, we checked, using simple linear regression analyses, that the expression values for more than 95.3% genes, which were predicted from estimate of fixed effects in the mixed model, were not in significant linear correlation with the estimates of the random and residual terms in the model.

**Figure 1:**
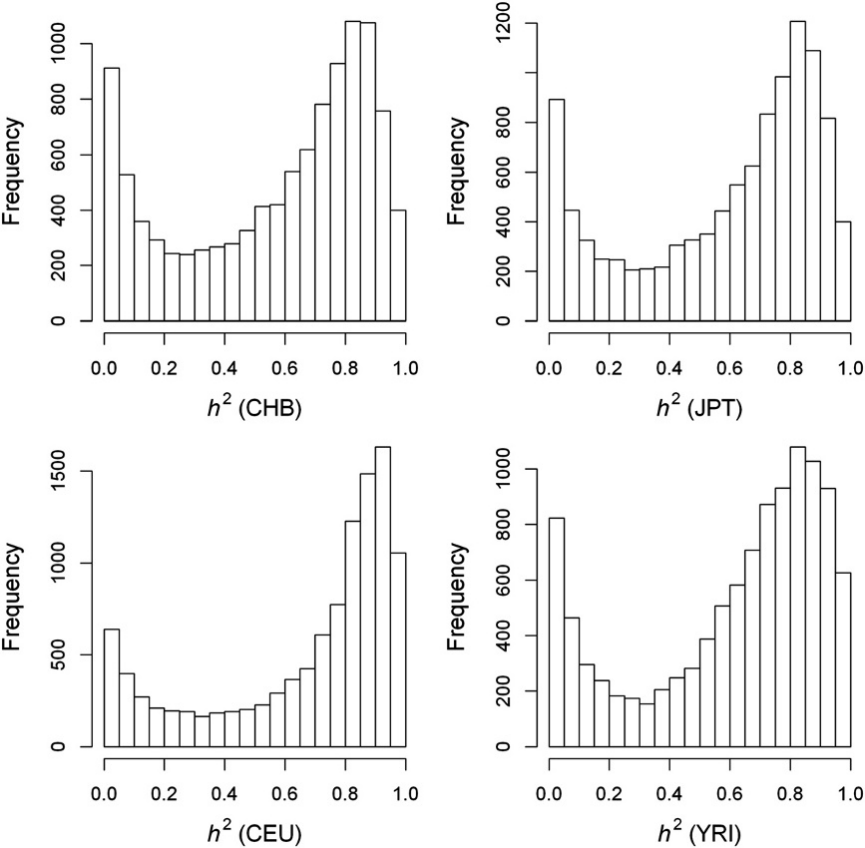
**Frequency distribution of gene expression heritability estimates (** ***h*** ^**2**^ **) in four HapMap populations.**

The four HapMap populations present a similar distribution and pattern of gene expression heritabilities, with one peak near 0 and another at about 90% (Figure 1). Pairwise comparison of heritability estimations between all HapMap populations further confirmed a strong correlation pattern (Pearson’s *r* > 0.89). Such a strong correlation of within-population heritability variability suggests either expression variability of the majority of the genes is under similar levels of constraints in all populations, or the *cis* or *trans* expression regulatory mechanisms of these genes have not undergone significant evolutionary divergence. Combining the four HapMap populations together, all genes showed heritability estimates larger than a nominal level of 0.1% and a similar heritability distribution pattern was observed (data not shown). In the following text, we focused on the analysis of the combined study sample from the four HapMap populations.

**Figure 2:**
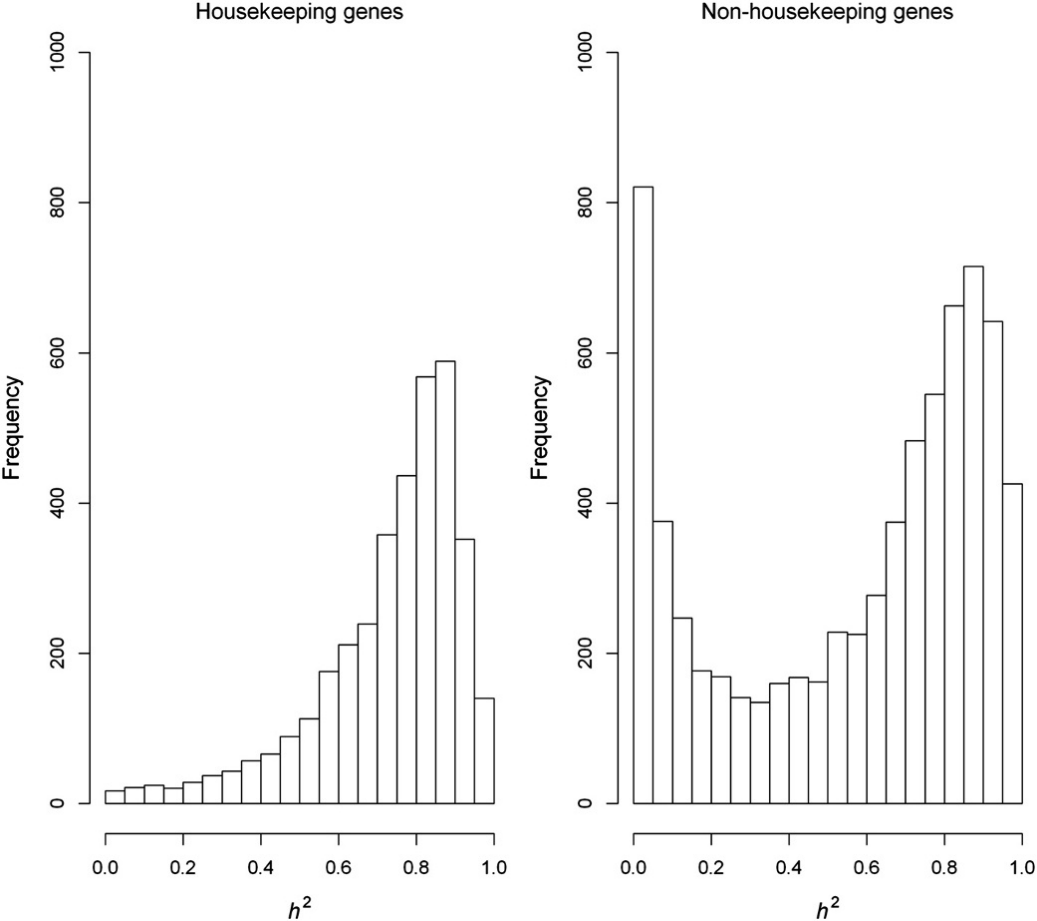
**Distribution of gene expression trait heritabilities in housekeeping and non-housekeeping genes.**

Genes can be characterized into constitutive (*i.e*. housekeeping) and tissue-specific groups according to whether they are ubiquitously expressed in all tissue/cell types for maintaining the fundamental requirement of basic cellular functions or specifically expressed to perform functions in differentiated tissues/cells. One recent study involving monozygotic twins reported that mean heritability of housekeeping gene expression is significantly greater than the mean for all genes [20]. With the samples of the unrelated individuals, we obtained similar finding in the present HapMap populations using a comprehensive housekeeping gene list of 6,909 genes derived from Zhu et al. [21] to partition genes into housekeeping (3,585 genes) and non-housekeeping (7,135 genes) categories. The housekeeping and non-housekeeping genes show distinct heritability distribution patterns (Figure 2), with the former presenting high levels of expression heritability, and the latter showing a bimodal pattern of expression heritability.

We explored the bimodal pattern of distribution of the heritability estimates for gene expression traits. Additional file 2 shows an empirical relationship between expression levels and heritabilities of gene expression traits. It is clear from the file that lower heritability estimates of gene expression (*h*^2^ < 0.2) were more likely to occur in genes with low expression levels, which is consistent with the fact that low expression levels are associated with a lower level of phenotypic variation. High heritability estimates (*h*^2^ > 0.5) were present in genes with a wider range of gene expression phenotype. Together with the fact that housekeeping genes had higher expression levels (median 8.29; mean 8.52) than non-housekeeping genes (median 7.46; mean 7.98), the results suggested a possible link between the expression levels and estimates of gene expression heritabilities. We will elaborate the observation in below.

### Genome-wide association eQTL analysis

In the genome-wide association scan, normalised gene expression levels were averaged among four replicated arrays for each individual and then scanned for genome-wide SNP associations using a multiple linear regression analysis with correction for population structure in the mixed HapMap populations. A total of 11,290 regression models involving 988 genes and 10,712 SNPs were declared significant at Bonferroni-corrected *P*-value threshold 3.85 × 10^-8^. It is noted that the number of genes was not taken into account in correction for multiple tests. At an overall false positive rate of 5%, about 550 expression-SNP models were expected to be significant at the given threshold. Because the 550 expected false positives accounted for only 4.7% of the total 11,290 discoveries, the present threshold should be recognized to be conservative and appropriate for further statistical analyses. For genes with multiple associations, the significant SNPs were merged into eQTLs. An eQTL in the present analysis was defined as an independent peak in the *P*-value profile across a given chromosome. Following Jiang et al. [22], any peak occurring within a chromosome region of 5 Mb in size was taken as a single eQTL peak. The eQTLs thus defined were further classified based on their physical distance from the associated gene, either as *cis* eQTLs if the SNP locates within 500 kb upstream of the transcript start and 500 kb downstream of 3’ end of the gene or otherwise as *trans eQTLs.* The 11,290 significant associations gave rise to 1,043 eQTLs, of which about two third (671) were *trans* eQTLs while only 372 were in *cis*, consistent with previous eQTL studies (for example, [7, 22]).

Previously, Stranger et al. [23] found an over-representation of *cis* associations (803 *cis* and 44 *trans*) from the same microarray dataset analysed here. We compared the *cis* eQTLs mapped in the present study with those predicted in the study of Stranger et al. and found that 591 out of 803 (73.6%) *cis* eQTL genes detected in Stranger et al. were included in the current selected gene set. More importantly, 252 *cis* eQTLs were common between the two studies as shown in Additional file 3, i.e., 70% of the eQTLs predicted here were also detected by Stranger et al., suggesting a high level of comparability of the present study to the previous eQTL analysis. Discrepancy in the number of eQTLs predicted between the two studies may be partly due to the fact that different genes or SNPs were selected for the analyses and partly due to different eQTL analysis methods used. Unlike the present analysis in which the population origin is used as a covariate to correct for structure in the linear regression analysis of the pooling sample, Stranger et al. [23] performed within population permutation to correct for inflated associations in simple linear regression. Because the population structure is clearly present in the pooled sample, linear regression conditional on the known population structure is much simpler but more effective in correcting for spurious associations than the permutation test. In fact, Stranger et al. [23] detected much less *trans* eQTLs largely due to their ways to determine the associated SNPs as the *trans* eQTLs. To avoid the computational burden and statistical challenges in testing all SNPs against all candidate expression traits, Stranger et al. tested for *trans* effects in only ~25,000 SNPs (roughly 1 percent of the total SNPs) selected for potential functional significance. In contrast, we screened for *trans* associations from the full SNP genotype data without prior selection.

**Figure 3:**
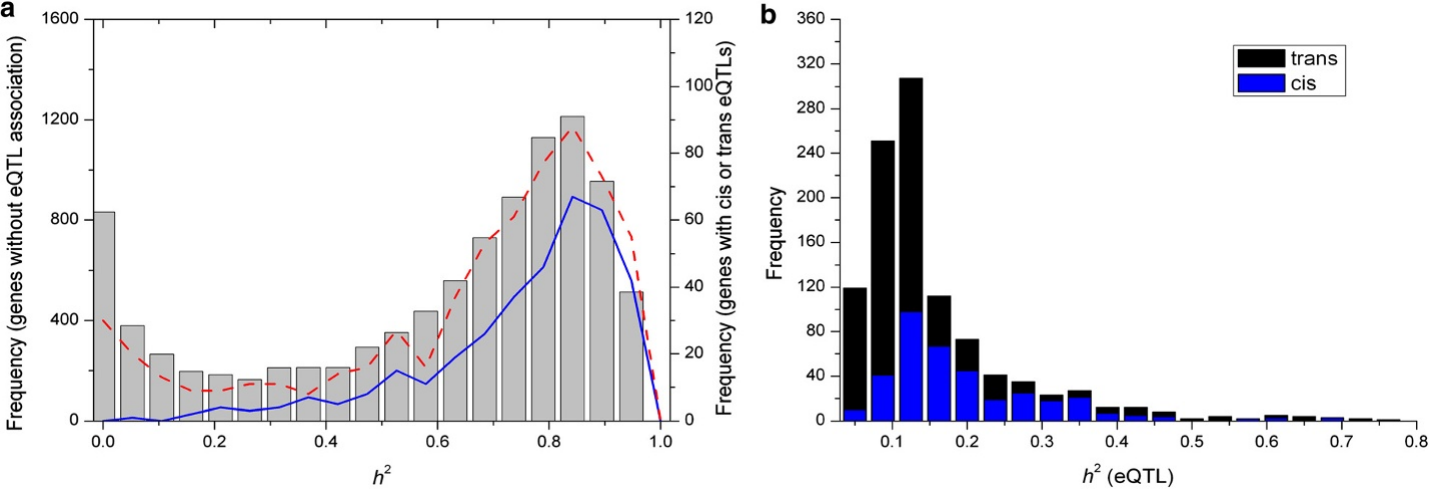
**Frequency distribution of heritability estimates (** ***h*** ^**2**^ **) in genes with or without significant eQTL associations. (a)** Histogram showing the distribution of heritability estimates of genome-wide gene expression levels. Grey bar, blue line and red dashed line present *h* ^2^ of genes without eQTL associations, genes with *cis* eQTLs and genes with only *trans* eQTLs, respectively. **(b)** Stacked histogram showing the distribution of the proportion of phenotypic variance explained by individual *cis* (blue) and *trans* (black) eQTLs.

Additional file 4 shows the chromosome locations of the eQTLs, with *cis* eQTLs indicated by the diagonal across each chromosome and *trans* eQTLs showing a strikingly uniformly distribution across the entire genome. Most genes with significant *cis* associations, presented only one *cis* eQTL (348 genes) while only 12 genes had two *cis* eQTLs. Eleven genes presented both *cis* and *trans* eQTLs, with each of them consisting of only one *cis* and one *trans* eQTLs. A complete list of predicted eQTLs is provided in Additional file 3. Most genes with strong *trans* associations have either one (620 genes) or two (16 genes) *trans* eQTLs. One notable gene RPS4P21 (ribosomal protein S4X pseudogene 21; Ensembl gene ID ENSG00000186008) at 34.5 Mb on the chromosome 19 presents associations with 13 *trans* eQTLs, 6 of which locate within a region from 91 to 241 Mb on chromosome 1. Partitioning genes into two groups according to presence or absence of eQTLs revealed that genes presenting eQTLs showed comparable heritability values (mean 71.6% and median 79.8%) to genes without eQTLs (mean 61.5% and median 72.6%), suggesting no apparent connection between the heritability levels and the predictability of eQTLs. Genes with eQTLs were further partitioned into those with *cis* eQTLs (including the eleven genes presenting both *cis* and *trans* eQTLs), and those with *trans* eQTLs only. This revealed that the genes with *trans* eQTLs shared a similar heritability distribution with genes lacking any eQTLs, *i.e.* these genes had low heritability levels, distinguishing them from genes with *cis* eQTLs which had distinctly higher heritability levels (Figure 3a).

### eQTL heritability

We were interested in how much heritability in gene expression traits could be accounted for by the predicted eQTLs. It is statistically challenging to directly estimate the proportion of phenotypic variance explained by each individual eQTL as the status of the underlying QTL gene linked to the SNP under study is unknown, so we followed an approximation approach as in Cockram et al. [24] by comparing the estimates of variance components between a SNP-inclusive mixed model (*i.e.* Equation 4) and a SNP-free model (*i.e.* Equation 1). The difference in the sum of variance component estimates can be used as an estimate of the phenotypic variance accounted for by the associated SNP. Here we used  to represent the estimate of the fraction of phenotypic variance explained by the eQTL. The sum of variance components is expected to decrease with the SNP-inclusive model because incorporation of additional explanatory variables generally improves model fit and hence will give a non-negative estimate of SNP heritability; to validate the present eQTL heritability estimation, we further confirmed that this decrease in the sum of variance components is due to a decrease in genetic variance components in the SNP-inclusive model relative to the SNP-free models and not due to a decrease in residual variance (Additional file 5). A stacked histogram presents the distribution of the heritability values for *cis* and *trans* eQTLs (Figure 3b). It is clear that the majority of the eQTLs explained individually very small fractions of phenotypic variance, particularly for the *trans* eQTLs, though there were some eQTLs explaining up to 80% of the phenotypic variance. Due to the large proportion of low heritability estimates among *trans* eQTLs, thus on average, this group tended to contribute smaller genetic variation (mean  = 15.6%) to gene expression variation compared with *cis* eQTLs (mean  = 22.0%).

We calculated heritability of expression phenotype by regressing midparent expression values on their offspring expression values for the 10,720 selected genes in the CEU and YRI trio population datasets [9]. After removing those pairs of individuals with predicted hidden relatedness, there are 28 (or 27) trio families retained in the CEU (or YRI) population. The microarray data from the trio families were pre-processed with the same normalisation procedure as described above for the unrelated individuals. Because every sample was replicated by four arrays, we took the average of replicates as gene expression estimate. In the trio-family analysis, heritability of gene expression was estimated by the regression coefficient Additional file 6 summarizes the heritability estimates, showing that there were about 32-35% genes showing negative heritability estimates in the CEU and YRI trio-family populations. The negative heritability estimates reflected nature of the regression analysis which is highly vulnerable to environment variation, particularly when a small population size is used. However, Fisher’s exact test demonstrated that the genes with the *cis* and *trans* eQTL genes predicted from the analysis above were specifically enriched for non-negative heritability estimates, suggesting a strong concordance between the trio-family based analysis and the population based eQTL analysis. We observed a highly significantly positive correlation in the expression heritability estimates between the two analyses (Pearson’s *r* = 0.22, *P* value < 10^-7^). Moreover, focus was on only those genes detected with significant eQTL regulation, the correlation coefficient increased to 0.31 (*P* value < 10^-7^). These may suggest that the mixed model analysis confers statistically more robust estimation of gene expression heritability, at least in the present setting.

### Missing heritability

**Figure 4:**
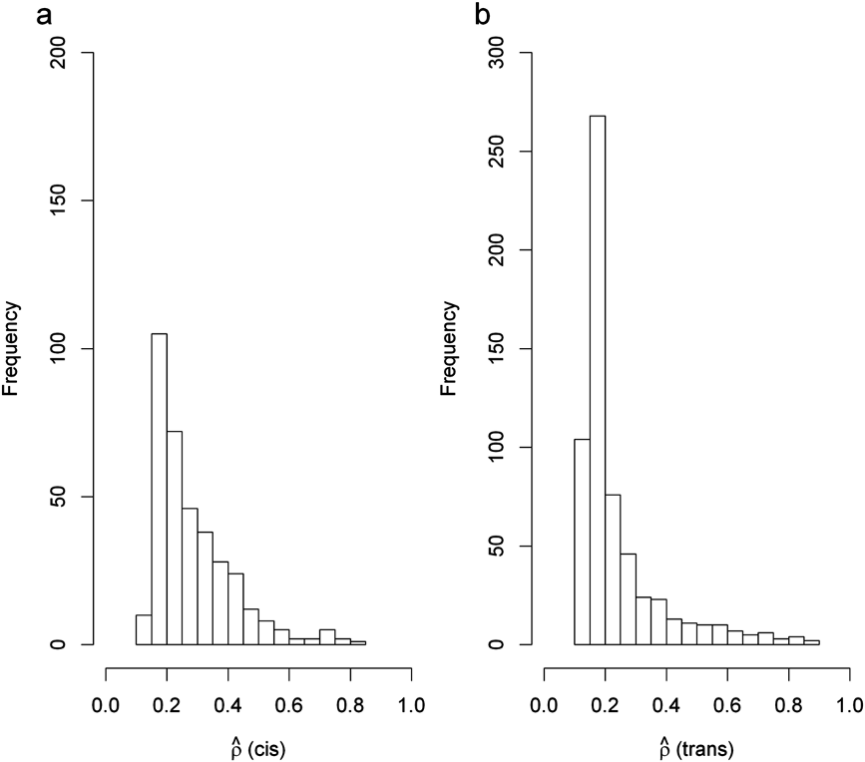
**Distribution of**  **values (** ***i.e.*** **, the proportion of genetic variation of gene expression traits accounted for by eQTLs) among 988 genes with significant eQTL associations. (a)** *cis* eQTLs, and **(b)** *trans* eQTLs.

For the 988 genes with significant eQTL associations, we were interested in the extent to which the genetic contributions to gene expression variation could be explained by the detected eQTLs. From a mathematical perspective, this is simply the ratio of heritability estimate at the eQTL (*i.e.*, ) or aggregated heritability from all detected eQTLs if multiple eQTLs are present, to the estimate of total heritability (*i.e.*, ) for a given gene, *i.e.*, . For simplicity, we denote this ratio by . For genes with multiple eQTLs, the aggregate heritability from all eQTLs was estimated by fitting genotypes from multiple eQTL peak SNPs into the linear mixed model and then following the same strategy used for single SNPs. In the first instance, multi-point genotypes were fitted in the linear mixed model in an additive form*, i.e.*, neglecting interaction terms. We summarized for each gene the aggregate heritabilities explained by the *cis* and/or *trans* eQTL components. The results (Figure 4a-b) show a right-skewed density distribution of the  values, *i.e.*, for the majority of the genes, only a small fraction of the total genetic variation could be explained by the detected *cis* or *trans* eQTLs, a phenomenon well-known as “missing heritability” in conventional complex trait analyses [1, 2]. The value of  varied widely between a minimum of 13.8% and a maximum of 88.8%. Although more *trans* eQTLs were predicted, the mean (median)  values were similar between *cis*: 28.44% (24.3%) and *trans*: 24.0% (17.6%) eQTLs.

To investigate the influence of gene-gene interactions on gene expression trait heritability, we selected genes presenting multiple eQTLs and for each selected gene we fitted a linear mixed model with incorporation of SNP-SNP multiplicative interaction effect. There were 39 genes with two eQTLs and 3 genes with three or more eQTLs. For simplicity, only genes presenting two eQTLs were selected for interaction test. As listed in Additional file 7, incorporation of interaction terms in the model in the model did not necessarily lead to an increased proportion of phenotypic variation for all the 39 genes tested. In 16 of the selected genes, the interaction terms had increased the explained genetic variance by as large as 35%. To assess the appropriateness of including interaction terms in the model, Akaike information criterion (AIC) was calculated for the models with or without interaction term. The models having a lower AIC value were recognized to be statistically more appropriate in decomposing the gene expression phenotypic variation. For five of the 39 genes, the models with the interaction term showed lower AIC values and hence were preferred in comparison with the corresponding additive models (Additional file 7). This demonstrates that the analysis of gene-gene interactions may be useful to uncover the genetic components that are not explained by additive gene effects only for some expression traits but this is not always the case for all genes.

## Discussion

Approaches that combine genome-wide gene expression profiling and genome-wide marker genotype data are offering new insights for dissecting the genetic basis of complex traits including common human diseases. In this study, we used publicly available datasets of microarray gene expression measurements and genome-wide SNPs from 210 HapMap individuals to examine the heritability of gene expression traits in LCL samples by using REML analysis of a variance component model. Differing from previous studies, which also analyzed the same microarray datasets but aimed to infer differentially expressed genes and/or to detect genetic controls of the expression regulations within or across ethnic groups (eg, [7–11, 23, 25–27]), this paper represents a detailed exploitation of the genome-wide distribution of heritabilities of gene expression traits. We present here for the first instance the *cis* and *trans* eQTLs through analysis using the information jointly from four HapMap populations. In contrast to a most recent study on gene expression heritability [12], in which the heritability in expression were estimated to be negative for a large number of genes, the present study developed a statistically robust variance component approach which may, ensure non-negative estimates of variance components and, in turn non-negative estimates of gene expression heritability in the range of 0 ~ 100%. The mixed model analysis was originally proposed for estimating quantitative genetic parameters using pedigree information of outbred populations [28]. The present variance component model utilises the relationship matrix inferred from genome-wide genotype, and the method developed here can be readily implemented for a population based analysis.

In this study, we observed a common pattern in distribution of gene expression heritabilities among four HapMap populations (CHB, JPT, CEU and YRI), suggesting possibly similar levels of constraints imposed on the expression variability of the most genes in the populations, or no apparent evolutionary divergence has been detected to impact the expression regulatory mechanisms of these genes. In this aspect, our result is consistent with a previous study which did also observe that expression variability of most human genes in one population was not markedly deviant from another population [29]. In the present study, we observed that heritability of gene expression was estimated to vary from as low as zero to as large as nearly 100%, indicating a large varying levels of genetic contribution to variation in the gene expression phenotype. While there was an abundance of genes with very low heritability, a large number of genes clustered at heritability levels of around 90%, resulting in a bimodal distribution. The analysis also shows that genes with larger variability tend to have a larger heritability (Additional file 8). In general, housekeeping genes exhibited greater heritability than non-housekeeping genes, demonstrating that a greater level of genetic control has been preserved in this group of genes. However, caution must be taken in interpreting the bimodality pattern in distribution of gene expression heritabilities. One of possible explanations for the bimodality is that a expression trait can be regulated either in *cis* or in *trans* and that the expression traits regulated in *cis* intend to have a higher heritability whilst the *trans* regulated expression traits intend to have a lower heritability. One other possibility as suggested from Additional file 2 is that those genes with an ultra low level of expressions are subjected to a very low statistical power to be detected for a significant genetic component in their regulation.

It needs to be stressed that the housekeeping genes listed here was derived by an independent study from multiple gene expression profiling experiments with different human tissues [21]. Therefore the different patterns of the heritability distribution between housekeeping and non-housekeeping genes revealed in this study should not be confounded by expression level related assignment of genes into different categories. Because information was not available for presence or absence of expression in the Illumina bead-based microarray dataset analysed, we have applied a filter to exclude lowly expressed genes to avoid the bias in estimates of gene expression heritability. Classifying genes into discrete abundance classes, e.g., highly expressed, lowly expressed and non-expressed, is not a simple task because genes show broad and quantitative expression levels with no clear separation into distinct classes with the current transcriptomic profiling platforms [30]. Many technique issues might influence the estimation of expression abundance, e.g., sensitivity of the platform, sample preparation and data processing algorithm. Recently, sequencing based application such as RNA-seq [31] is increasingly being used to quantify gene expression with more precise measurement. However, the RNA-seq technique has its own limitations. For example, sequencing depth exerts a profound impact on the expression abundance estimation and the subsequent data analyses such as differential expression prediction [32]. We argue that care must be taken interpreting the gene expression data. Nevertheless, we noted that RNA-seq data sets for LCLs of two of the present HapMap populations were publicly available [33, 34]. We downloaded the normalised RNA-seq expression data for YRI and CEU LCL samples and confirmed that about 88% and 85% of the 10,720 currently selected genes were present in the RNA-seq datasets for YRI and CEU populations, respectively. This result supports the appropriateness of the current strategy to select expressed genes for the eQTL analysis from the microarray data.

With a genome-wide scan of gene-SNP associations, 1,043 eQTLs were detected for 988 genes. Concordant with previous studies (e.g., [7, 22]), we obtained an excess of *trans* over *cis* eQTLs. Moreover, we demonstrated in Additional file 9 that genes with or without eQTLs had a similar distribution in expression levels, removing the concern that gene expression levels had substantial impact on prediction of eQTLs. We estimated the fractions of genetic variations explained by eQTLs by using a simple approach which compares the variance component estimates between SNP-free and SNP-inclusive models. Compared to methods that require calculating allelic effect and allele frequency at individual locus (e.g., [6]), the present eQTL heritability estimation approach provides a simple alternative and allows multiple eQTLs to be jointly analyzed for both additive and interactive effects. We demonstrated that both categories of eQTL cumulatively explained comparable proportions of the total heritability despite that *trans* eQTLs individually have weaker effects than *cis* eQTLs, a result consistent with other eQTL studies [35]. While heritability may be considered to evidence the genetic control of phenotypic variation (gene expression level), it is an obvious assumption that higher heritability implies a greater chance to map genetic variants responsible for variation in gene expression. However, we have shown that this assumption may be true for *cis* eQTLs but not for *trans* eQTLs, because while genes with *cis* eQTLs aggregated at high heritability levels, genes with *trans* eQTLs shared a similar heritability distribution to that of genes with no eQTLs at all. One possible explanation is that *trans* effects in LCL could be introduced by non-genetic variation of *in vitro* factors, which could mimic *trans* regulation but does not resemble true biologically heritable genetic regulation. Another potential reason is the lack of power to detect *trans* eQTLs in those genes without eQTLs in the present sample given stringent significance threshold and the small genetic effects conferred by the *trans* eQTLs. The implication of this finding is that heritability should not be used as a filter to screen genes for the detection of eQTLs.

A number of explanations have been suggested for the failure of associated variants to fully explain heritability in conventional complex trait association analyses; these include a lack of statistical power to detect loci with minor effects, the existence of interactions between genetic factors and/or between genes and environment, and the possibility of influence by epigenetic factors; the first of these explanations has been extensively studied [4]. In this study, we investigated gene-gene interaction (epistasis) as a potential source to account for unexplained heritability in genetic association studies. Genetic studies have long identified specific instances of genetic interactions in model species [e.g., [36]]. A recent study in yeast *Saccharomyces cerevisiae* had confirmed interactions partially explained the heritability of complex traits missed by the additive genetic contributions [37]. However, carrying out genome-wide interaction analysis in human is still not feasible because the prevalence of interactions in human dataset is still largely unknown [38, 39]. We take advantage of the fact that while predicting genetic interactions *a priori* from population data may be difficult computationally and of low power, it is much more straightforward to detect epistasis among variants *a posteriori* once they have been detected [38]. For a number of genes with multiple eQTLs, we modelled multiplicative interaction effects and evaluated the contribution of gene-gene interactions to total gene expression heritability. Epistatic effects were detected in 5 genes through AIC model selection, leading to a substantial increase in the genetic variance explained by 10 ~ 35%. However, it needs to be emphasized that the number of genes predicted with multiple eQTLs could be underestimated possibly due to the small sample size and/or over-stringent significance threshold implemented in the association test. It would be certainly likely to detect such eQTLs when the significance confidence was lowered but this may enforce concern of the type 1 error. Hence the present finding about *cis* and *trans* eQTL interactions is clearly subject to variation due to use of small samples. Nonetheless, our results clearly show that the interaction is a crucial term for exploring and unravelling the mystery of missing heritability at gene expression level.

## Conclusions

We implemented a variance component analysis for inferring the proportion of phenotypic variations explained by genetic factors for genome-wide gene expression traits from unrelated individuals. The study reveals that heritability of the genome-wide gene expression traits varies from 100% to almost zero which is common between four HapMap populations, that the *cis*- regulating expression traits usually have a larger heritability than *trans*- regulating expression traits, and that distribution of the expression traits heritability differs between the house-keeping and non-house keeping genes. The study illustrates that interaction between eQTLs contributes significantly to a missed fraction of heritability in the expression traits.

### Availability

Computer program implementing the present variance component model analysis is available in Additional file 10. The microarray gene expression data analysed in this paper were downloaded from the Gene Expression Omnibus (GEO) database (http://www.ncbi.nlm.nih.gov/geo) through accession number GSE6536 [9].

## Electronic supplementary material

Additional file 1: **Multidimensional scaling plot of the HapMap populations using the first and second principal coordinates (PCo) (a) and the first and third PCo (b).** (DOC 283 KB)

Additional file 2: **The relationship between heritability estimates and mean expression levels in four HapMap populations.** (DOC 472 KB)

Additional file 3: ***Cis*** **- and** ***trans*** **- eQTLs detected in the mixed HapMap populations.** (XLS 201 KB)

Additional file 4: **Chromosome locations of predicted eQTLs in the HapMap populations.** (DOC 517 KB)

Additional file 5: **Changes in variance component estimates between SNP-free (equation** 1**) and SNP-inclusive (equation** 4**) linear mixed model analyses.** (DOC 217 KB)

Additional file 6: **Summary of the midparent-offspring regression analysis in CUE and YRI trio populations.** (DOC 246 KB)

Additional file 7: **Heritability and phenotypic variance explained by the eQTLs for genes presenting two eQTLs.** (DOC 76 KB)

Additional file 8: **Relationship between heritability and variability of gene expression levels.** (DOC 276 KB)

Additional file 9: **Mean gene expression levels for genes with** ***cis*** **eQTLs,** ***trans*** **eQTLs and no eQTLs, respectively.** (DOC 219 KB)

Additional file 10: **R program source code implementing the present linear mixed model analysis.** (ZIP 85 KB)
