# Supplementary material for: Genome-wide eQTLs and heritability for gene expression traits in unrelated individuals
Source: BMC Genomics. 2014 Jan 9;15(1):13. doi: 10.1186/1471-2164-15-13 (PMC4028055; doi:10.1186/1471-2164-15-13)
Supplement: Supplementary file 1 — Additional file 1: Multidimensional scaling plot of the HapMap populations using the first and second principal coordinates (PCo) (a) and the first and third PCo (b). (DOC 283 KB) [file 12864_2013_6999_MOESM1_ESM.doc]

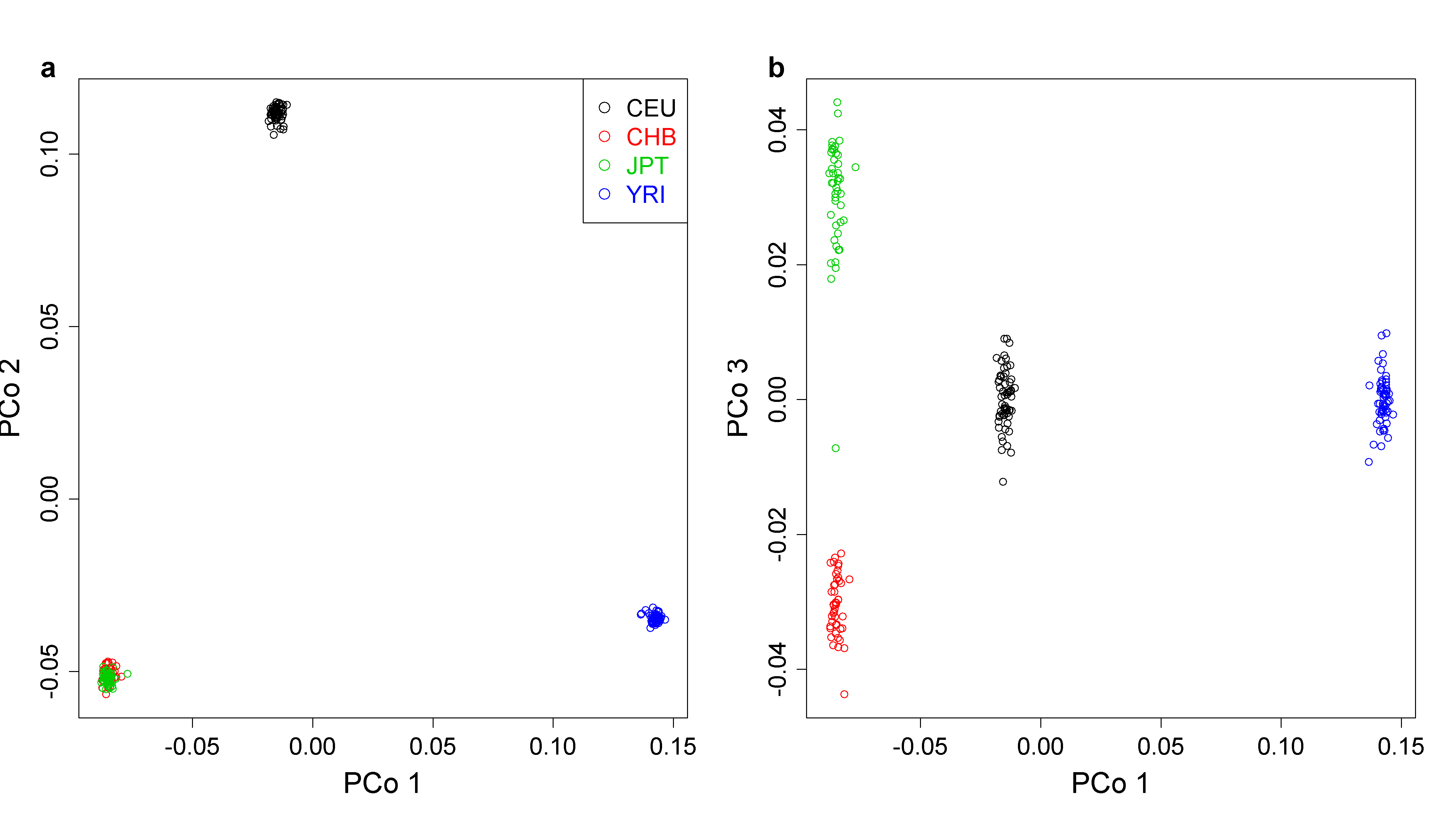


Additional file 1: Multidimensional scaling plot of the HapMap populations using the first and second principal coordinates (PCo) (a) and the first and third PCo (b).
