# Supplementary material for: Genome-wide eQTLs and heritability for gene expression traits in unrelated individuals
Source: BMC Genomics. 2014 Jan 9;15(1):13. doi: 10.1186/1471-2164-15-13 (PMC4028055; doi:10.1186/1471-2164-15-13)
Supplement: Supplementary file 2 — Additional file 2: The relationship between heritability estimates and mean expression levels in four HapMap populations. (DOC 472 KB) [file 12864_2013_6999_MOESM2_ESM.doc]

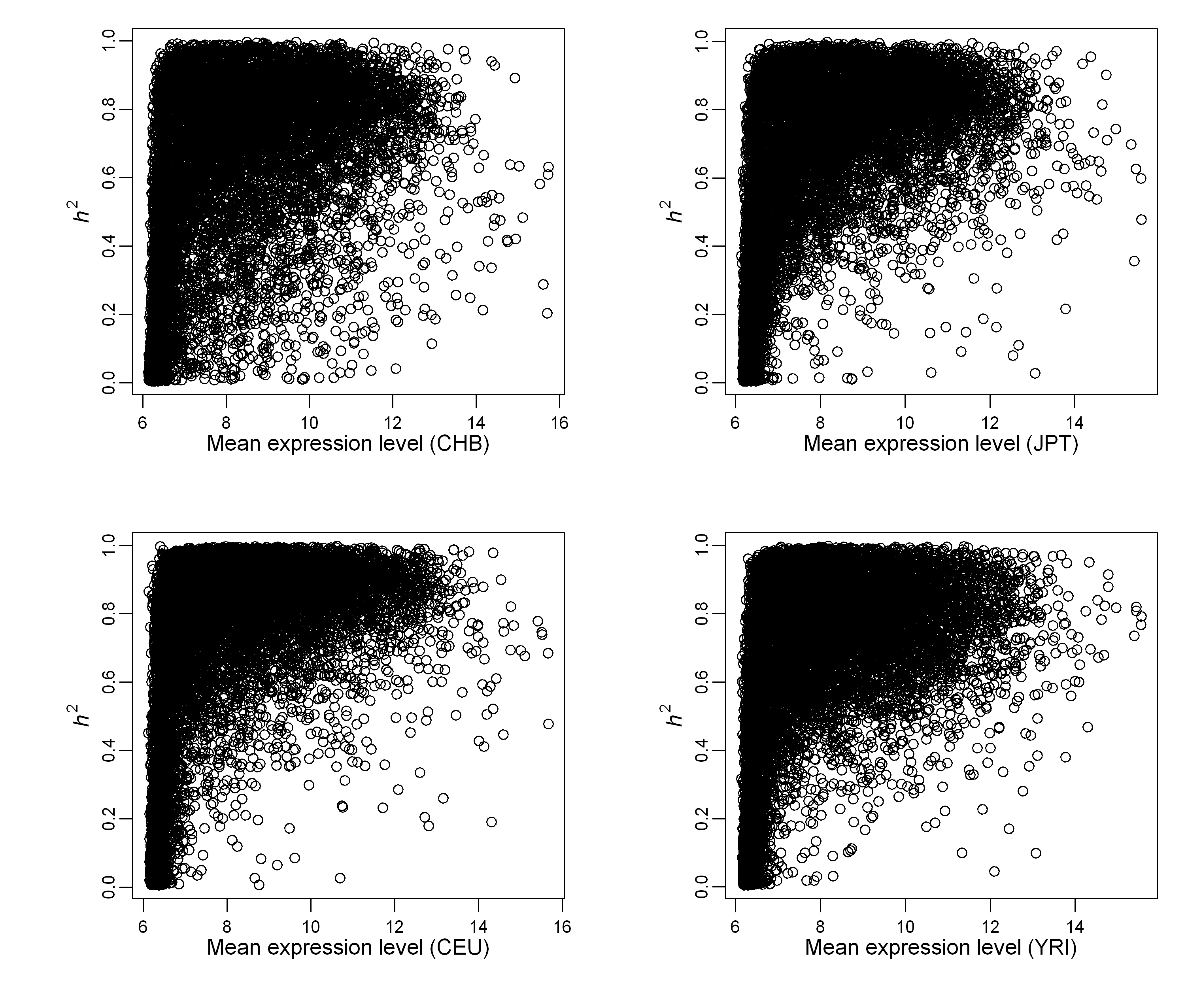


Additional file 2: The relationship between heritability estimates and mean expression levels in four HapMap populations.
