# Supplementary material for: Genome-wide eQTLs and heritability for gene expression traits in unrelated individuals
Source: BMC Genomics. 2014 Jan 9;15(1):13. doi: 10.1186/1471-2164-15-13 (PMC4028055; doi:10.1186/1471-2164-15-13)
Supplement: Supplementary file 5 — Additional file 5: Changes in variance component estimates between SNP-free (equation 1) and SNP-inclusive (equation 4) linear mixed model analyses. (DOC 217 KB) [file 12864_2013_6999_MOESM5_ESM.doc]

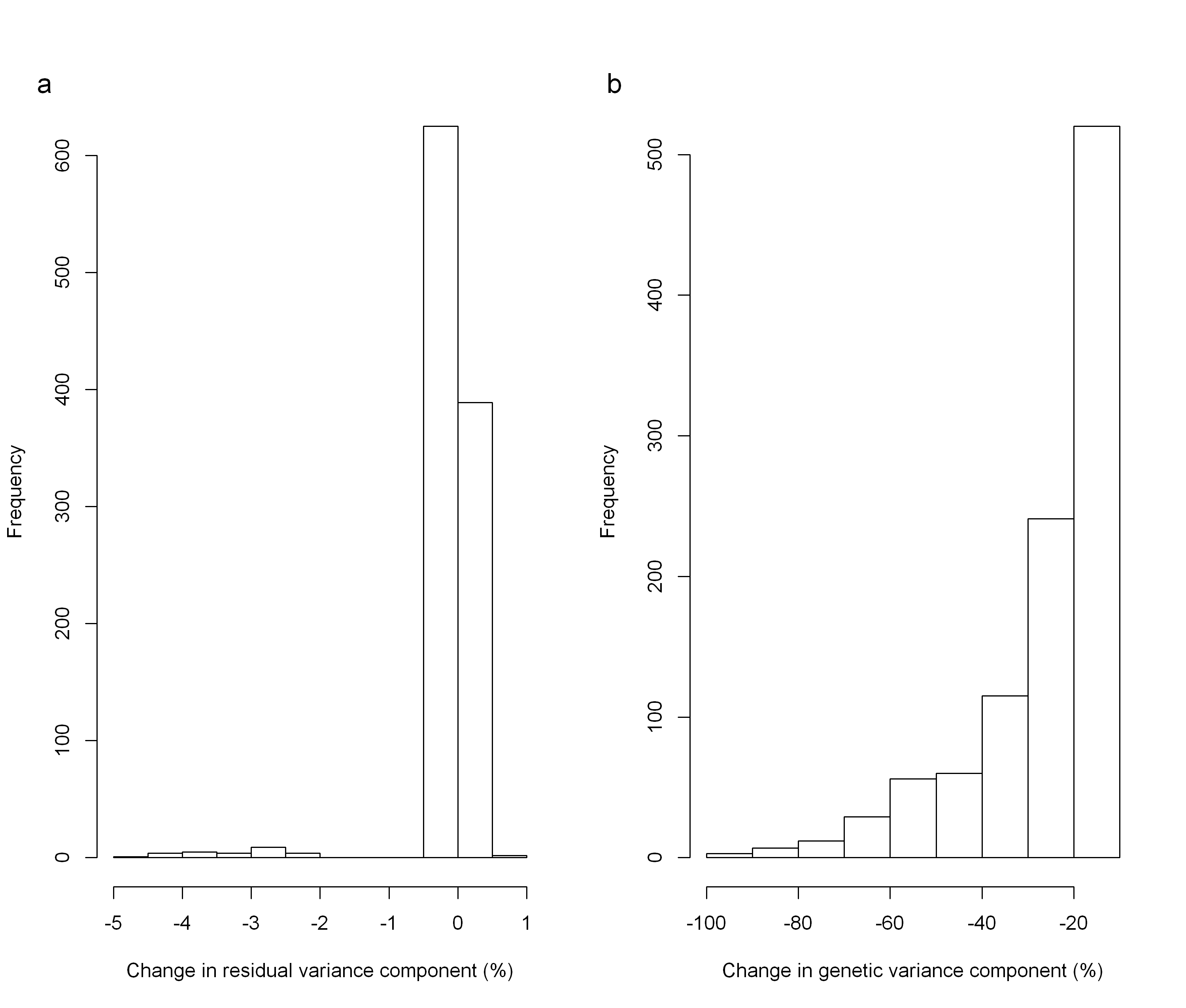


Additional file 5: Changes in variance component estimates between SNP-free (equation 1) and SNP-inclusive (equation 4) linear mixed model analyses. The x-axis is$(\hat{\hat{\sigma}}_{e}^{2}-\hat{\sigma}_{e}^{2})\times100/\hat{\sigma}_{e}^{2}$ in (a) and $(\hat{\hat{\sigma}}_{g}^{2}-\hat{\sigma}_{g}^{2})\times100/\hat{\sigma}_{g}^{2}$ in (b).
