# Supplementary material for: Genome-wide eQTLs and heritability for gene expression traits in unrelated individuals
Source: BMC Genomics. 2014 Jan 9;15(1):13. doi: 10.1186/1471-2164-15-13 (PMC4028055; doi:10.1186/1471-2164-15-13)
Supplement: Supplementary file 6 — Additional file 6: Summary of the midparent-offspring regression analysis in CUE and YRI trio populations. (DOC 246 KB) [file 12864_2013_6999_MOESM6_ESM.doc]

a.
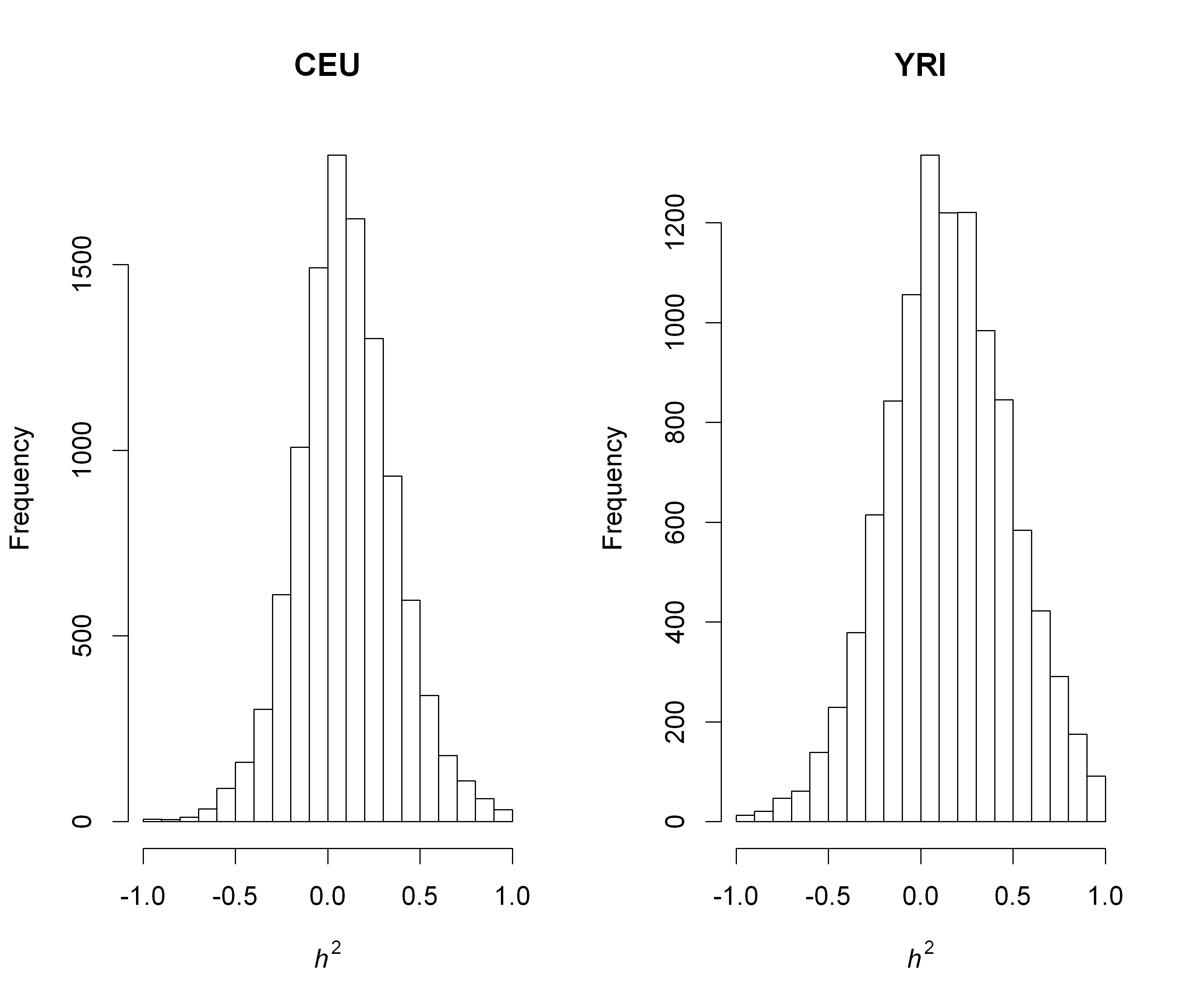


b.

|  | CEU | | | YRI | | |
| --- | --- | --- | --- | --- | --- | --- |
|  | *h*^2^ ≥ 0 | *h*^2^ < 0 | P-value^*^ | *h*^2^ ≥ 0 | *h*^2^ < 0 | P-value^*^ |
| *cis* eQTL genes | 313 | 47 | < 2.2 × 10^-16^ | 321 | 39 | < 2.2 × 10^-16^ |
| *trans* eQTL genes | 471 | 168 | 4.9 × 10^-6^ | 469 | 170 | 2.7 × 10^-3^ |
| All genes | 6,994 | 3,726 | - | 7,301 | 3,419 | - |

**Additional file 6: Summary of the midparent-offspring regression analysis in CUE and YRI trio populations.** a, distribution of gene expression trait heritabilities for 10,720 selected genes in the CEU and YRI trios; b, the number of genes having negative or non-negative heritability estimates.

^*^P-value was obtained from Fisher’s exact test of whether eQTL genes were specifically enriched for non-negative heritability estimates than random chance.
