# Supplementary material for: Genome-wide eQTLs and heritability for gene expression traits in unrelated individuals
Source: BMC Genomics. 2014 Jan 9;15(1):13. doi: 10.1186/1471-2164-15-13 (PMC4028055; doi:10.1186/1471-2164-15-13)
Supplement: Supplementary file 7 — Additional file 7: Heritability and phenotypic variance explained by the eQTLs for genes presenting two eQTLs. (DOC 76 KB) [file 12864_2013_6999_MOESM7_ESM.doc]

Additional file 7: Heritability and phenotypic variance explained by the eQTLs for genes presenting two eQTLs.

| # | Gene | | eQTLs | | | $\hat{h}_{trait}^{2}$ (%) | $\hat{\rho}$*^a^*  (%) | $\hat{\rho}$*^b^*  (%) | △AIC |
| --- | --- | --- | --- | --- | --- | --- | --- | --- | --- |
|  | Ensembl ID | Chr | #*cis* | eQTL 1 SNP (chr:position) | eQTL 2 SNP (chr:position) |  |  |  |  |
| 1 | ENSG00000197747 | 1 | 0 | rs2932574 (1:150248644) | rs7077814 (10:67636066) | 82.3 | 34.7 | 34.6 | 8 |
| 2 | ENSG00000171806 | 1 | 0 | rs10748557 (10:82409749) | rs178940 (17:31086799) | 46.8 | 18.2 | 18.2 | 8.7 |
| 3 | ENSG00000116132 | 1 | 0 | rs524252 (1:74505185) | rs10945820 (6:162713399) | 89.9 | 19.7 | 19.6 | 7.7 |
| 4 | ENSG00000143653 | 1 | 0 | rs7526237 (1:244964757) | rs6699332 (1:245001634) | 77.3 | 22.3 | 22.1 | 10.3 |
| 5 | ENSG00000115239 | 2 | 0 | rs1921998 (2:217520622) | rs12590252 (14:56082358) | 92.2 | 26.9 | 29.8 | -1.5 |
| 6 | ENSG00000106780 | 9 | 0 | rs10818474 (9:122529785) | rs2900219 (9:126147101) | 89.7 | 26.9 | 26.6 | 8.2 |
| 7 | ENSG00000232936 | 10 | 0 | rs1328914 (9:13044470) | rs8085710 (18:63584734) | 86.4 | 29.7 | 29.4 | 7.7 |
| 8 | ENSG00000166268 | 12 | 0 | rs6553967 (4:177623194) | rs11177845 (12:68462901) | 51.0 | 33.2 | 34.1 | 5.1 |
| 9 | ENSG00000134864 | 13 | 0 | rs837344 (13:99984057) | rs837313 (13:100003429) | 38.8 | 16.6 | 16.4 | 10 |
| 10 | ENSG00000125952 | 14 | 0 | rs7711670 (5:38144655) | rs4785919 (16:2917263) | 81.1 | 16.3 | 16.0 | 8.9 |
| 11 | ENSG00000125124 | 16 | 0 | rs17355357 (16:54958477) | rs9889162 (16:55113289) | 97.7 | 54.8 | 55.4 | 3.1 |
| 12 | ENSG00000075643 | 18 | 0 | rs1540052 (4:103357000) | rs1941458 (18:32079742) | 79.8 | 32.3 | 33.7 | 1.3 |
| 13 | ENSG00000205396 | 19 | 0 | rs17277762 (7:80551140) | rs16975256 (13:109510340) | 98.1 | 41.1 | 55.5 | -50.3 |
| 14 | ENSG00000053438 | 20 | 0 | rs6821225 (4:147222168) | rs11218832 (11:122191931) | 94.9 | 33.4 | 35.7 | 1.7 |
| 15 | ENSG00000160284 | 21 | 0 | rs4325512 (15:80094724) | rs9637215 (21:46420663) | 49.7 | 16.8 | 20.6 | -8.3 |
| 16 | ENSG00000128342 | 22 | 0 | rs13037480 (20:40365271) | rs7286517 (22:46796207) | 21.1 | 12.1 | 11.9 | 11.8 |
| 17 | ENSG00000116874 | 1 | 1 | rs12032374 (1:119385360) | rs2436384 (5:156553029) | 80.8 | 20.3 | 21.0 | 5.8 |
| 18 | ENSG00000151806 | 4 | 1 | rs16857402 (4:44401210) | rs12590252 (14:56082358) | 86.8 | 31.3 | 31.0 | 8.7 |
| 19 | ENSG00000186470 | 6 | 1 | rs11098914 (4:128356851) | rs9358933 (6:26471667) | 76.7 | 30.9 | 31.0 | 7.8 |
| 20 | ENSG00000124613 | 6 | 1 | rs3856206 (1:162012453) | rs980961 (6:27468998) | 91.0 | 26.5 | 26.3 | 7.1 |
| 21 | ENSG00000169919 | 7 | 1 | rs4718278 (7:64881031) | rs7791814 (7:65002924) | 34.9 | 16.8 | 16.5 | 12.2 |
| 22 | ENSG00000155158 | 9 | 1 | rs11942462 (4:176199680) | rs10118629 (9:15286450) | 82.6 | 28.6 | 28.2 | 8.2 |
| 23 | ENSG00000196072 | 10 | 1 | rs12241379 (10:102042363) | rs199771 (20:22329961) | 85.9 | 16.1 | 15.8 | 7.9 |
| 24 | ENSG00000074319 | 11 | 1 | rs12419063 (11:18481894) | rs11652559 (17:33076712) | 86.4 | 34.6 | 34.6 | 7.9 |
| 25 | ENSG00000108523 | 17 | 1 | rs10879192 (12:39471471) | rs238245 (17:4788188) | 59.9 | 19.0 | 18.8 | 8 |
| 26 | ENSG00000108599 | 17 | 1 | rs533134 (1:225356762) | rs2042046 (17:19959433) | 92.0 | 34.0 | 34.6 | 4.4 |
| 27 | ENSG00000125821 | 20 | 1 | rs2161425 (5:166241585) | rs6045469 (20:18494812) | 84.0 | 22.5 | 22.3 | 10.1 |
| 28 | ENSG00000162441 | 1 | 2 | rs6661806 (1:9960055) | rs7528979 (1:10016044) | 72.6 | 21.2 | 25.2 | -4.8 |
| 29 | ENSG00000116985 | 1 | 2 | rs580699 (1:39979097) | rs2138686 (1:40015516) | 84.4 | 32.8 | 33.1 | 7.8 |
| 30 | ENSG00000116791 | 1 | 2 | rs10890142 (1:74951717) | rs1327090 (1:75039067) | 95.9 | 26.0 | 25.6 | 7 |
| 31 | ENSG00000183891 | 2 | 2 | rs7572167 (2:19964469) | rs10197385 (2:20040055) | 95.5 | 17.3 | 16.9 | 7.7 |
| 32 | ENSG00000115902 | 2 | 2 | rs4671631 (2:64998291) | rs1035165 (2:65013100) | 90.5 | 21.5 | 21.7 | 6.7 |
| 33 | ENSG00000151470 | 4 | 2 | rs4975274 (4:129997526) | rs1709421 (4:130278429) | 49.7 | 16.5 | 16.5 | 9.5 |
| 34 | ENSG00000196284 | 6 | 2 | rs609643 (6:44813281) | rs7745506 (6:45137988) | 69.9 | 20.5 | 20.3 | 8.6 |
| 35 | ENSG00000112096 | 6 | 2 | rs9355742 (6:159997163) | rs5746136 (6:160023074) | 60.3 | 23.2 | 24.1 | 6.1 |
| 36 | ENSG00000158623 | 7 | 2 | rs10863 (7:129932825) | rs2288081 (7:130007836) | 91.2 | 17.2 | 18.0 | 4.3 |
| 37 | ENSG00000166321 | 10 | 2 | rs6480671 (10:74570789) | rs12256735 (10:75020904) | 85.6 | 33.8 | 33.6 | 8 |
| 38 | ENSG00000173915 | 10 | 2 | rs7913461 (10:104991315) | rs6580 (10:105196864) | 97.5 | 28.4 | 34.9 | -11 |
| 39 | ENSG00000179889 | 16 | 2 | rs4985167 (16:14990366) | rs1741 (16:15037852) | 81.5 | 20.7 | 20.6 | 7.8 |

*^a^*, the fraction of genetic variance explained by additive effect of eQTLs

*^b^*, the fraction of genetic variance explained by additive and multiplicative (interaction) effects of eQTLs

*#cis*, number of *cis* eQTLs.

△AIC, difference in Akaike information criterion (AIC) values between models with and without interaction term. The model with interaction term is preferred relative to that without interaction term if △AIC value is negative, and rejected otherwise.
