# Supplementary material for: Genome-wide eQTLs and heritability for gene expression traits in unrelated individuals
Source: BMC Genomics. 2014 Jan 9;15(1):13. doi: 10.1186/1471-2164-15-13 (PMC4028055; doi:10.1186/1471-2164-15-13)
Supplement: Supplementary file 8 — Additional file 8: Relationship between heritability and variability of gene expression levels. (DOC 276 KB) [file 12864_2013_6999_MOESM8_ESM.doc]

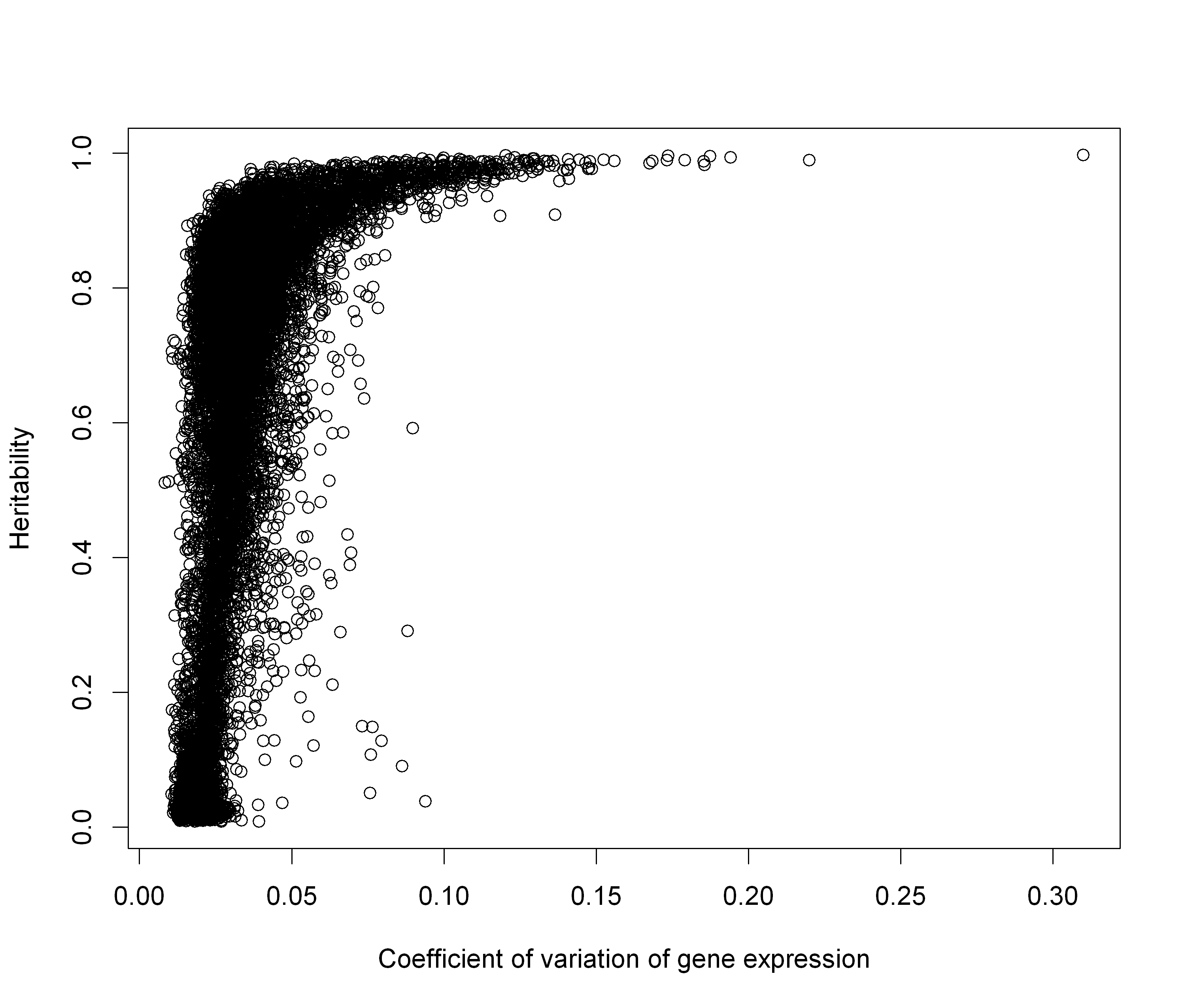


Additional file 8: Relationship between heritability and variability of gene expression levels. Coefficient of variation is calculated as standard deviation of gene expression phenotype divided by the mean.
