# Supplementary material for: Genome-wide eQTLs and heritability for gene expression traits in unrelated individuals
Source: BMC Genomics. 2014 Jan 9;15(1):13. doi: 10.1186/1471-2164-15-13 (PMC4028055; doi:10.1186/1471-2164-15-13)
Supplement: Supplementary file 9 — Additional file 9: Mean gene expression levels for genes with cis eQTLs, trans eQTLs and no eQTLs, respectively. (DOC 219 KB) [file 12864_2013_6999_MOESM9_ESM.doc]

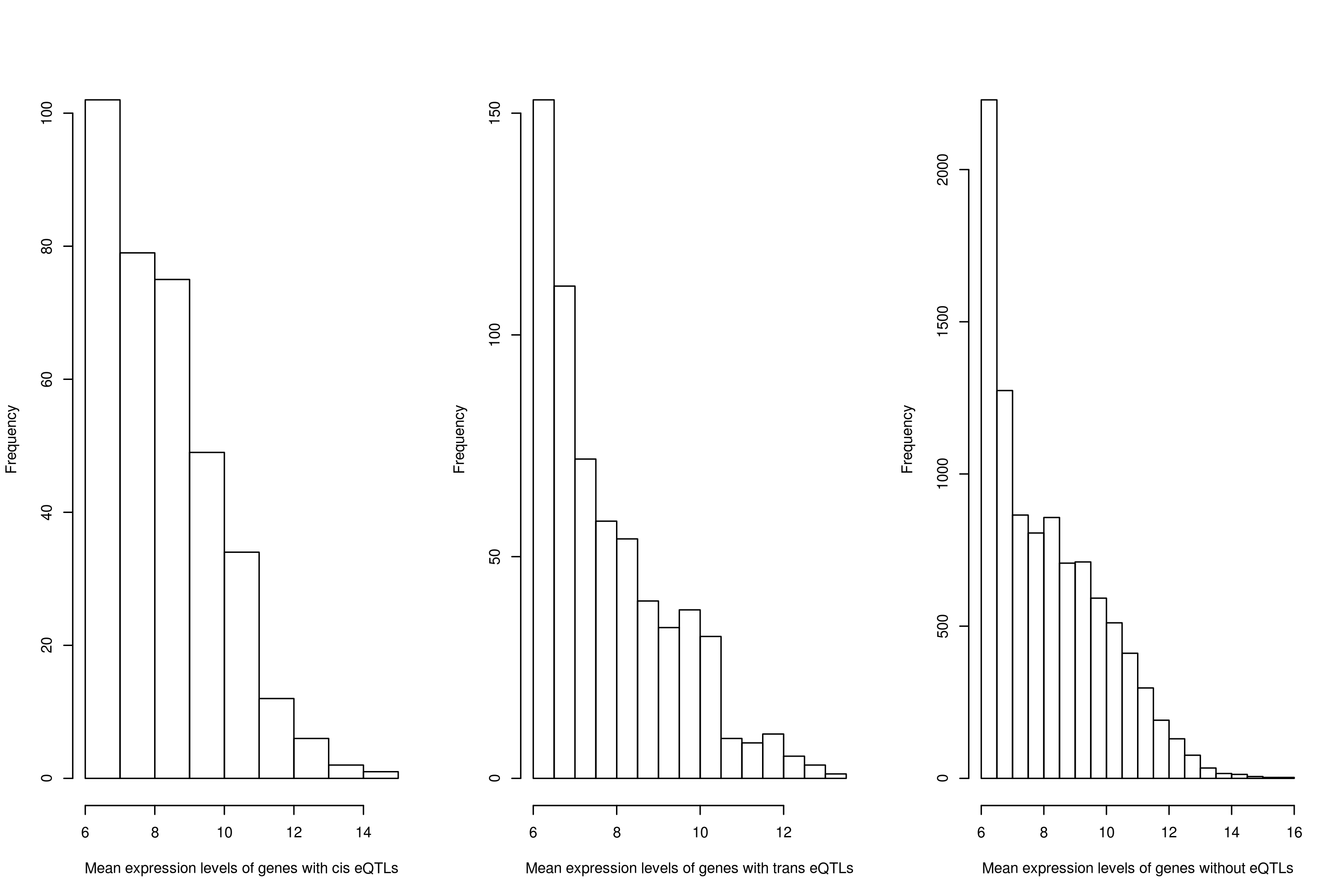


Additional file 9: Mean gene expression levels for genes with *cis* eQTLs, *trans* eQTLs and no eQTLs, respectively.
