# Supplementary material for: Genome-wide eQTLs and heritability for gene expression traits in unrelated individuals
Source: BMC Genomics. 2014 Jan 9;15(1):13. doi: 10.1186/1471-2164-15-13 (PMC4028055; doi:10.1186/1471-2164-15-13)
Supplement: Supplementary file 10 — Additional file 10: R program source code implementing the present linear mixed model analysis. (ZIP 85 KB) [file 12864_2013_6999_MOESM10_ESM.zip › readme.pdf]

We implemented the Restricted Maximum Likelihood (REML) and Maximum Likelihood (ML) analyses of the present variance component model in an R script (amvce.R). To use this script, you must first install R (http://www.r-project.org), source this script in the R session and then call function amvce. In the following, we give a brief description of the usage of the function amvce. At the end of this documentation, an example is provided for fitting a variance component model for gene expression heritability analysis using a sample dataset.

Usage

amvce(fixed, data, random, correlation, method, initials=NULL, tolerance=1.0e-3, maxIter=100, disp=FALSE, ...)

Arguments

fixed	a two-sided linear formula object describing the fixed-effects part of the model, with the response on the left of a ~ operator and the terms, separated by + operators, on the right.
data	an optional data frame containing the variables named in fixed and random. By default the variables are taken from the environment from which amvce is called.
random	optionally, a one-sided formula of the form ~ x1, specifying for the model the random effects. An optional relationship structure between levels of the random effects can be provided using argument correlation.
correlation	optionally, a list with two named elements: A (a matrix describing the correlation structure of random effects, with rownames and colnames matching the levels of the random factors, if argument random is specified) and R (a matrix of correlation structure for residual terms). In a typical quantitative genetic model, A is a genetic relationship matrix. By default, both A and R are identity matrices.
method	optionally, a character string. There are three methods available: AI-REML (REstricted Maximum Likelihood by Average Information), EM-REML (REML

	by Expectation Maximization), and EM-ML (Maximum Likelihood by Expectation Maximization). The default method is AI-REML.
initials	optionally, a vector specifying the starting values of the genetic variance and residual variance components for the REML or ML iterations.
tolerance	optionally, tolerance for the convergence criterion in the amvce optimization algorithm. Default is 1.0e-3.
maxIter	optionally, maximum number of iterations for the amvce optimization algorithm. Default is 100.
disp	optionally, a logical value specifying whether to print real time status.
...	additional arguments. Not used currently.

Value

an object of class amvce representing the model parameter estimations. The amvce object includes the following components.

call	a list containing an image of the amvce call that produced the object.
coefficients	a vector containing the estimates of fixed effects.
VC	a vector containing estimates of genetic and residual variance components.
apVar	an approximate covariance matrix for the variance component estimates.
apVb	an approximate covariance matrix for the fixed effect estimates.
loglik	logarithmic likelihood of the model.
loglik0	logarithmic likelihood of the reduced model (without genetic variance component).

References

The model formulation is described in Harville (1977). The expectation maximization algorithm follows the general framework of Searle et al (1992) and the average information algorithm is obtained from Johnson and Thompson (1995).

1. Harville DA: **Maximum Likelihood Approaches to Variance Component Estimation and to Related Problems**. *Journal of the American Statistical Association* 1977, **72**(358):320-338.
2. Searle SR, Casella G, McCulloch CE: **Variance Components**: John Wiley & Sons, Inc; 1992.
3. Johnson DL, Thompson R: **Restricted Maximum Likelihood Estimation of Variance Components for Univariate Animal Models Using Sparse Matrix Techniques and Average Information**. *Journal of Dairy Science* 1995, **78**(2):449-456.

Example

```
#Change the working directory to where contains the amvce.R script and example data
> setwd('/path/to/script')
#read example data from file example/hmm26479.RData
> load('example/hmm26479.RData')
#This example dataset includes a data.frame called dh, which contains normalised expression
# values for gene hmm26479 in 206 HapMap individuals, and a matrix called grm, which
# contains pairwised IBD relationships between the 206 HapMap individuals.
> head(dh) #check the gene expression data
> grm[1:5,1:5] #check part of the genetic relationship matrix
#load the script amvce.R into the R session
> source('amvce.R')
#fit linear mixed model
> ( fit=amvce(hmm26479~POP,data=dh,random=~IID, correlation=(A=grm))  )
```
